# Variations in Testing for HIV and Other Sexually Transmitted Infections Across Gender Identity Among Transgender Youth

**DOI:** 10.1089/trgh.2018.0047

**Published:** 2019-02-20

**Authors:** Akshay Sharma, Erin Kahle, Kieran Todd, Sarah Peitzmeier, Rob Stephenson

**Affiliations:** ^1^ Department of Health Behavior and Biological Sciences, School of Nursing, University of Michigan, Ann Arbor, Michigan.; ^2^ Center for Sexuality and Health Disparities, School of Nursing, University of Michigan, Ann Arbor, Michigan.; ^3^ Department of Systems, Population and Leadership, School of Nursing, University of Michigan, Ann Arbor, Michigan.

**Keywords:** gender identity, HIV testing, minority health, STI testing, transgender youth

## Abstract

**Purpose:** Transgender youth are at high risk for human immunodeficiency virus (HIV) and other sexually transmitted infections (STIs), but their rates of screening are unknown. This study sought to quantify HIV and other STI testing levels and to examine variations in testing levels across three categories of gender identity: transgender men, transgender women, and nonbinary individuals.

**Methods:** Between June 2017 and June 2018, 186 transgender youth aged 15–24 years were recruited into a randomized trial of home HIV testing supplemented with telehealth-based counseling. Information on sociodemographics, health care utilization, sexual activity, stress and resilience, and history of HIV and other STI testing was obtained. Multivariable logistic regression models were formulated to identify variations in testing for HIV and other STIs across gender identities.

**Results:** Twenty-eight of 186 participants (15.1%) reported testing for HIV in the past year, and 42 (22.6%) reported testing for other STIs. Transgender women were less likely to have been tested for HIV (adjusted odds ratio [aOR]: 0.15, 95% confidence interval [CI]: 0.03–0.78) and other STIs (aOR: 0.33, 95% CI: 0.11–0.99), but nonbinary individuals were equally likely to have been tested compared with transgender men. Participants who agreed that their health care provider is knowledgeable about transgender health issues were thrice as likely to have been tested for HIV (aOR: 3.29, 95% CI: 1.36–7.97) and other STIs (aOR: 3.05, 95% CI: 1.40–6.63) compared with those who disagreed.

**Conclusion:** Low levels of testing among transgender youth highlight the exigency of improving gender- and age-appropriate HIV and other STI prevention services. Given that provider knowledge of transgender health issues was strongly associated with testing, training health care providers in transgender-related care could prove beneficial.

## Introduction

Transgender persons are estimated to comprise 0.4% of the US population,^1^ but represent a diverse community with a broad spectrum of sexual identities and health-influencing behaviors. Unfortunately, they have poorer mental and physical health outcomes compared with their cisgender counterparts.^2^ Estimates for the percentage of youth in the United States who identify as transgender vary between 0.2%^3^ and 1.3%.^4^ Transgender youth have unique needs, and may experience gender minority stressors such as discrimination, rejection, nonaffirmation of gender identity, internalized transphobia, and anticipated stigma during their developmental years.^5^ Transgender youth may also face barriers to accessing and receiving health care services, such as economic marginalization and social stigma, contributing to gender- and age-related health disparities.^6^ Latest research indicates that transgender youth are at high risk for depression, self-harm, and suicide,^7^ as well as human immunodeficiency virus (HIV) and other sexually transmitted infections (STIs).^8^

Recent statistics from the Centers for Disease Control and Prevention (CDC) highlight the disproportionate burden of HIV among transgender individuals. Among 3 million HIV testing events reported to the CDC in 2015, the percentage of transgender persons who received a new diagnosis was more than three times the national average.^9^ From 2009 to 2011, high levels of HIV positivity were identified in transgender women (2.7% of 13,154), followed by cisgender men (0.9% of 4,534,426), transgender men (0.5% of 2,364), and cisgender women (0.2% of 4,753,672).^10^ Data from the National HIV Surveillance System (NHSS) indicate that of the 2,351 transgender individuals with newly diagnosed HIV during 2009–2014, 84.0% were transgender women, 15.4% were transgender men, and 0.7% had another gender identity.^11^ More than one-third of the diagnoses among 1,974 transgender women were in ages 13–24 years (8.3% aged 13–19 years and 28.0% aged 20–24 years), and more than one-fifth among 361 transgender men were in that age group (6.4% aged 13–19 and 16.6% aged 20–24).

To date, studies to quantify the burden of other STIs such as gonorrhea, chlamydia, syphilis, genital herpes, and human papillomavirus among transgender individuals have mostly involved small convenience samples.^12^ An analysis of 292 transgender person-visits to a municipal clinic in San Francisco, California between 2006 and 2009 estimated an STI prevalence ranging from 2.0% to 11.1%.^13^ Another retrospective study that analyzed the electronic health records of 145 sexually active transgender persons who attended an adolescent and young adult urban community health center in Boston, Massachusetts between 2001 and 2010, reported an STI prevalence ranging from 1.4% to 2.8%.^14^ Not all federal, state, and local agencies in the United States collect or have complete data on transgender persons, precluding the ability to obtain population-based STI estimates among transgender youth.

Most of the existing sexual health research among gender minorities, including that on HIV and other STI prevention, focuses on transgender women.^15,16^ Although some studies prioritizing transgender men have been conducted in developed countries,^17,18^ few data are available for nonbinary individuals.^19^ These people may not identify with any gender or feel like they exist between or outside the binary notions of male or female gender. Regardless of gender identity or sexual orientation, the same factors promoting HIV acquisition and transmission, such as having multiple sex partners and engaging in condomless sex, can also increase the risk for other STIs. Testing is the first step in offering pre-exposure prophylaxis (PrEP) to those who test HIV-negative, or initiating treatment for HIV and other STIs among those who test positive. Understanding testing behaviors among transgender youth, particularly within different categories of gender identity, is essential for designing effective prevention interventions.

Nationwide rates of testing for HIV and other STIs among gender minority persons are currently limited. A recent analysis of 2014–2015 Behavioral Risk Factor Surveillance System (BRFSS) data from 27 US states and Guam revealed that only 10.0% of 732 transgender women and 10.2% of 451 transgender men reported testing for HIV in the past year.^20^ Percentages for nonbinary individuals were not reported because of small sample size, and data for transgender adolescents were not available because BRFSS only surveys individuals ≥18 years old.

Given the paucity of information on HIV and other STI testing behaviors among adolescent and young transgender persons, this study seeks to fill an important gap in the extant prevention literature. The primary purpose of this analysis is to quantify levels of testing for HIV and other STIs in the past year among transgender youth aged 15–24 years and to examine variations in screening across three categories of gender identity: transgender men, transgender women, and nonbinary individuals either assigned female at birth (AFAB) or assigned male at birth (AMAB). The secondary purpose is to describe their recent sexual activity (anal, vaginal, or oral), as well as summarize their gender-related stress and resilience levels.

## Methods

### Overview of study design

Project Moxie is a randomized trial involving transgender youth in the United States that seeks to examine whether the addition of telehealth-based counseling to rapid home HIV testing can create gains in HIV testing over a 6-month follow-up period (ClinicalTrials.gov registration No. NCT03185975). Participants first complete a baseline survey, after which they are randomized to one of two study arms. Those in the experimental arm receive an oral fluid rapid home HIV test kit, and test with a remotely located counselor during a prescheduled video conferencing session. Those in the control arm also receive an oral fluid rapid home HIV test kit, but test on their own and report their results via the study's website. Follow-up assessments are conducted through online surveys at 3 months, and at 6 months. The research protocol was approved by the Institutional Review Board at the University of Michigan (eResearch ID No. HUM00102906), and has been described in detail elsewhere.^21^ Results presented in this article are a cross-sectional analysis of data from the baseline survey. The trial was ongoing at the time of submitting this article, but results pertaining to whether the addition of telehealth-based counseling to rapid home HIV testing improves screening rates, reduces sexual risk behaviors, and enhances linkage to care for newly diagnosed HIV-positive transgender youth will be published shortly.

### Participant recruitment procedures

Participants were recruited between June 2017 and June 2018 using advertisements and postings placed on the following social media platforms: Facebook, Instagram, Twitter, Craigslist, and Tumblr. Recruitment advertisements featured photos of people with varying gender identities and call-to-action text directing interested individuals to the Project Moxie website. Eligibility criteria included being 15–24 years of age, currently residing in the United States, self-identifying as non-cisgender, never having been diagnosed with HIV, willing to receive a rapid home HIV test kit at an address of their choice, and having access to a computer, smartphone, or tablet that can support VSee, a HIPAA secure video conferencing software.^22^

### Baseline survey measures

To elicit participants' current gender identity, the survey included a question with the following response options: “male/man,” “female/woman,” “trans male/trans man/trans masculine,” “trans female/trans woman/trans feminine,” “genderqueer/gender nonconforming,” “agender/gender fluid,” and “gender not listed.” Demographic information collected included age, race/ethnicity, educational level, employment status, sexual orientation, current living situation, history of homelessness, and health insurance coverage. Participants were also asked about the extent to which they agree that the health care provider they most frequently visit is knowledgeable about transgender health issues. Responses were collected as a 5-point Likert item (“strongly agree,” “somewhat agree,” “neither agree nor disagree,” “somewhat disagree,” and “strongly disagree”). Gender-related stress and resilience was measured using scales to assess the interrelated but distinct domains of discrimination, rejection, nonaffirmation of gender identity, internalized transphobia, anticipated stigma, and community connectedness.^23^ Participants were also asked if they have ever been forced to have sexual contact with someone against their will. Information on alcohol, tobacco, and other drug use in the past 3 months was collected. Questions on recent sexual activity included the number of anal, vaginal, and oral sex partners in the past 3 months, sexual positioning (insertive or receptive), and condom use during each encounter. Data were obtained on HIV testing history, including whether participants had ever been tested, and if so, the time, location, type, and result of their most recent HIV test, as well as their frequency of annual testing, similar to the authors' previous work with sexual minority men.^24^ The survey included questions on previous home HIV testing, perceived barriers to using a home HIV test kit, PrEP awareness, and current PrEP use. Participants were also asked about other STIs they had been tested for in the past year.

### Descriptive analyses and modeling

SAS version 9.4 (Cary, NC) was used to conduct statistical analyses. The analytic sample was restricted to participants who provided information on their HIV and other STI testing history, as well as recent sexual activity (anal, vaginal, or oral). Fisher's exact tests were used to compare characteristics of these individuals with those who were excluded. Due to small numbers, responses to the question on current gender identity were combined to construct a new variable with three categories: transgender men (including “male/man,” “trans male/trans man/trans masculine”), transgender women (including “female/woman,” “trans female/trans woman/trans feminine”), and nonbinary individuals (including “genderqueer/gender nonconforming,” “agender/gender fluid,” and “gender not listed”). However, HIV and other STI testing levels among nonbinary individuals were also examined based on their sex assigned at birth (i.e., AFAB or AMAB).

Demographic and behavioral characteristics of the sample were summarized using descriptive statistics. Details on participants' sexual activity in the past 3 months were also tabulated. Scores for the gender-related stress and resilience scales were calculated for each participant and summarized, overall and stratified by testing for HIV and other STIs in the past year. Kruskal–Wallis tests were conducted to assess differences in these scores across categories of testing behaviors. Data on the HIV testing characteristics of participants who reported ever having been tested were also tabulated.

Two multivariable logistic regression models were formulated to examine variations in testing for HIV and other STIs in the past year across different categories of gender identity. Both models were adjusted for age, race/ethnicity, educational level, the extent to which participants agree that their health care provider is knowledgeable about transgender health issues, whether they had consumed alcohol, tobacco products, or other drugs in the past 3 months, and number of sex partners in the past 3 months. Condition indices and variance decomposition proportions were examined to check for potential multicollinearity. Results are presented as adjusted odds ratios (aORs) with 95% confidence intervals (CIs).

## Results

Overall, 1,113,955 advertising impressions resulted in 33,182 (3.0%) clicks to the study's website during the recruitment period. Of these, 2,707 (8.1%) provided electronic informed consent, of whom 1,365 (50.4%) started the eligibility screener. Six hundred ninety-eight (25.8%) met the eligibility criteria, of whom 480 (68.8%) proceeded to create an online account. Of the 216 (45.0%) who registered by providing legitimate contact information (name, email, phone number, and shipping address), 202 (93.5%) started the baseline survey. Of these, 11 (5.4%) did not provide information on their HIV or other STI testing history, and 5 (2.5%) did not answer any questions on recent sexual activity. The analytic sample was restricted to the remaining 186 (92.1%) participants. No statistically significant demographic differences were observed between these individuals and those excluded due to missing data.

Descriptive characteristics are presented in Table 1. Seventy-five of 186 participants (40.3%) were categorized as transgender men (6 “male/man,” 69 “trans male/trans man/trans masculine”), 33 (17.7%) as transgender women (4 “female/woman,” 29 “trans female/trans woman/trans feminine”), and 78 (41.9%) as nonbinary individuals (46 “genderqueer/gender nonconforming,” 24 “agender/gender fluid,” and 8 “gender not listed”). Fifty-four of 78 (69.2%) nonbinary individuals were AFAB, and 24 (30.8%) were AMAB. The average age was 19 years, approximately two-thirds (65.6%) were non-Hispanic white, and more than half (53.8%) were currently in or had graduated from high school. The majority were working or studying (80.6%), living with their parents (50.5%), and had some kind of health insurance (87.1%). Less than one-third (30.7%) agreed that the health care provider they most frequently visit is knowledgeable about transgender health issues, and more than two-thirds (70.4%) had not accessed any medical interventions to affirm their gender. Almost a quarter (23.1%) reported ever being forced to have sexual contact with someone against their will. More than two-thirds (71.5%) had consumed alcohol, tobacco products, or other drugs (mostly cannabis) in the past 3 months. Fifteen (20.0%) transgender men, 2 (6.1%) transgender women, 6 (11.1%) nonbinary AFAB individuals, and 5 (20.8%) nonbinary AMAB individuals reported testing for HIV in the past year. Twenty-two (29.3%) transgender men, 6 (18.2%) transgender women, 6 (11.1%) nonbinary AFAB individuals, and 8 (33.3%) nonbinary AMAB individuals reported testing for other STIs in the past year. Regarding PrEP awareness and use, 103 (55.4%) had heard about it, and 1 (0.5%) was currently on PrEP.

**Table 1.** Demographic and Behavioral Characteristics of 186 Transgender Youth, United States, June 2017 to June 2018

	Gender identity			
Characteristic	Transgender men (*N*=75), *n* (%)	Transgender women (*N*=33), *n* (%)	Nonbinary individuals^a^ (*N*=78), *n* (%)	Total (*N*=186), *n* (%)
Age group (years)^b^				
15–18	39 (52.0)	8 (24.2)	39 (50.0)	86 (46.2)
19–24	36 (48.0)	25 (75.8)	39 (50.0)	100 (53.8)
Race/ethnicity				
Non-Hispanic white	52 (69.3)	19 (57.6)	51 (65.4)	122 (65.6)
Non-Hispanic non-white^c^	17 (22.7)	6 (18.2)	16 (20.5)	39 (21.0)
Hispanic	6 (8.0)	8 (24.2)	11 (14.1)	25 (13.4)
Educational level				
Ninth grade to high school graduate/GED	45 (60.0)	14 (42.4)	41 (52.6)	100 (53.8)
Some college/technical school or higher	30 (40.0)	19 (57.6)	37 (47.4)	86 (46.2)
Employment status				
Exclusively working^d^	25 (33.3)	16 (48.5)	20 (25.6)	61 (32.8)
Exclusively studying	22 (29.3)	7 (21.2)	29 (37.2)	58 (31.2)
Both working and studying^e^	11 (14.7)	4 (12.1)	16 (20.5)	31 (16.7)
Unemployed	17 (22.7)	6 (18.2)	13 (16.7)	36 (19.4)
Sexual orientation				
Queer	25 (33.3)	10 (30.3)	36 (46.2)	71 (38.2)
Bisexual	21 (28.0)	8 (24.2)	17 (21.8)	46 (24.7)
Homosexual/gay	10 (13.3)	7 (21.2)	9 (11.5)	26 (14.0)
Other^f^	19 (25.3)	8 (24.2)	16 (20.5)	43 (23.1)
Current living situation				
Own house or apartment	25 (33.3)	12 (36.4)	18 (23.1)	55 (29.6)
Parent's house or apartment	37 (49.3)	10 (30.3)	47 (60.3)	94 (50.5)
Other^g^	13 (17.3)	11 (33.3)	13 (16.7)	37 (19.9)
Ever been homeless				
No	60 (80.0)	26 (78.8)	64 (82.1)	150 (80.7)
Yes	15 (20.0)	7 (21.2)	14 (18.0)	36 (19.4)
Health insurance coverage				
Uninsured	11 (14.7)	3 (9.1)	10 (12.8)	24 (12.9)
Insured^h^	64 (85.3)	30 (90.9)	68 (87.2)	162 (87.1)
Health care provider is knowledgeable about transgender health issues^i^				
Disagree	51 (68.0)	19 (57.6)	59 (75.6)	129 (69.4)
Agree	24 (32.0)	14 (42.4)	19 (24.4)	57 (30.7)
Accessed any medical interventions to affirm their gender				
No	40 (53.3)	19 (57.6)	72 (92.3)	131 (70.4)
Yes^j^	35 (46.7)	14 (42.4)	6 (7.7)	55 (29.6)
Ever been forced to have sexual contact with someone against their will				
No	56 (74.7)	26 (78.8)	61 (78.2)	143 (76.9)
Yes	19 (25.3)	7 (21.2)	17 (21.8)	43 (23.1)
Consumed alcohol, tobacco products, or other drugs in the past 3 months				
No	17 (22.7)	13 (39.4)	23 (29.5)	53 (28.5)
Yes^k^	58 (77.3)	20 (60.6)	55 (70.5)	133 (71.5)
No. of sex partners in the past 3 months^l^				
0	24 (32.0)	11 (33.3)	20 (25.6)	55 (29.6)
1	34 (45.3)	12 (36.4)	34 (43.6)	80 (43.0)
≥2	17 (22.7)	10 (30.3)	24 (30.8)	51 (27.4)
Tested for HIV in the past year				
No^m^	60 (80.0)	31 (93.9)	67 (85.9)	158 (85.0)
Yes	15 (20.0)	2 (6.1)	11 (14.1)	28 (15.1)
Tested for other STIs in the past year				
No	53 (70.7)	27 (81.8)	64 (82.1)	144 (77.4)
Yes^n^	22 (29.3)	6 (18.2)	14 (18.0)	42 (22.6)

^a^ Includes 54 who were assigned female at birth and 24 who were assigned male at birth.

^b^ Mean=19 years, median=19 years.

^c^ Includes 12 non-Hispanic black/African American, 7 Asian, 2 Native American/Alaskan Native, 1 Middle Eastern, and 17 mixed.

^d^ Includes 42 exclusively working part-time, and 19 exclusively working full-time.

^e^ Includes 26 working part-time and studying, 3 working full-time and studying, and 2 working both full-time and part-time.

^f^ Includes 16 pansexual, 12 questioning/unsure, 9 heterosexual/straight, 2 asexual, 2 demisexual, 1 polysexual, and 1 sexually fluid.

^g^ Includes 14 living in a non-family member's house or apartment, 11 living in another family member's house or apartment, 6 living in a college dorm, 2 living in a room and board, halfway house, or shelter/welfare hotel, 2 living on the street, 1 living in a boarding school, and 1 living in a sober home.

^h^ Includes 77 with private/work-based insurance, 45 with Medicaid/Medicare, 11 with school-based insurance, 1 with Veterans Administration benefits, 19 with some other insurance, and 9 with multiple kinds of insurance.

^i^ Responses collected as a 5-point Likert item reflecting the extent to which participants agree with this statement. Disagree includes 27 who “strongly disagree,” 48 who “somewhat disagree,” and 54 who “neither agree nor disagree.” Agree includes 22 who “strongly agree,” and 35 who “somewhat agree.”

^j^ Includes 6 who reported taking pubertal blockers, 53 who reported taking hormones, and 24 who reported undergoing some kind of surgical procedure, of whom 2 reported undergoing genital reconstruction surgery (numbers are not mutually exclusive).

^k^ Includes 113 who consumed alcohol, 52 who used tobacco products, and 86 who used other drugs, of whom 81 reported using cannabis, 9 reported using synthetic opioids, 9 reported using amphetamines, 8 reported using cocaine, 7 reported using sedatives, 2 reported using hallucinogens, and 2 reported using amyl nitrites (numbers are not mutually exclusive).

^l^ Summarizes data from 180 who responded to questions on anal sex, 123 who responded to questions on vaginal sex, and 121 who responded to questions on oral sex.

^m^ Includes 141 who reported never having been tested.

^n^ Includes 34 who reported testing for gonorrhea and chlamydia, 24 who reported testing for syphilis, 22 who reported testing for genital herpes, and 22 who reported testing for human papillomavirus (numbers are not mutually exclusive).

GED, General Equivalency Development; HIV, human immunodeficiency virus; STI, sexually transmitted infection.

Seven in 10 participants (*n*=131, 70.4%) reported having some kind of sexual encounter (anal, vaginal, or oral) in the past 3 months. Data on specific sexual activities are summarized in Table 2. Fifty-three of 180 participants (29.4%) who responded to the questions on anal sex, 91 of 123 (74.0%) who responded to the questions on vaginal sex, and 108 of 121 (89.3%) who responded to the questions on oral sex reported having at least 1 partner in the past 3 months. Of the 53 participants who engaged in anal sex, 38 (71.7%) reported condomless anal sex, and of those, 5 (13.2%) reported testing for HIV, and 7 (18.4%) reported testing for other STIs in the past year. Of the 91 participants who engaged in vaginal sex, 35 (38.5%) reported condomless vaginal sex, and of those, 6 (17.1%) reported testing for HIV, and 7 (20.0%) reported testing for other STIs in the past year. Of the 108 participants who engaged in oral sex, 78 (72.2%) reported condomless oral sex, and of those, 11 (14.1%) reported testing for HIV, and 15 (19.2%) reported testing for other STIs in the past year.

**Table 2.** Sexual Activity in the Past 3 Months Reported by 186 Transgender Youth, United States, June 2017 to June 2018

Characteristic	Anal sex, *n* (%)	Vaginal sex, *n* (%)	Oral sex, *n* (%)
Overall	*N*=180	*N*=123	*N*=121
No. of sex partners			
0	127 (70.6)	32 (26.0)	13 (10.7)
1	35 (19.4)	71 (57.7)	76 (62.8)
≥2	18 (10.0)	20 (16.3)	32 (26.4)
Subsets reporting at least one sex partner	*N*=53	*N*=91	*N*=108
No. of partners with whom engaged in condomless sex			
0	15 (28.3)	56 (61.5)	30 (27.8)
1	23 (43.4)	29 (31.9)	59 (54.6)
≥2	15 (28.3)	6 (6.6)	19 (17.6)
Had insertive sex^a^ with:			
0 partners	22 (41.5)	50 (54.9)	108 (100.0)
≥1 partners with a condom	11 (20.8)	10 (11.0)	0 (0.0)
≥1 partners without a condom	20 (37.7)	31 (34.1)	0 (0.0)
Had receptive sex^b^ with:			
0 partners	9 (17.0)	25 (27.5)	18 (16.7)
≥1 partners with a condom	12 (22.6)	57 (62.6)	12 (11.1)
≥1 partners without a condom	32 (60.4)	9 (9.9)	78 (72.2)

^a^ Insertive sex was described to participants as “you put your genitals into a partner's anus, vagina, or mouth.”

^b^ Receptive sex was described to participants as “a partner's genitals were put into your anus, vagina, or mouth.”

Regarding gender-related stress and resilience, 163 participants (87.6%) responded to all items in each scale, 12 (6.5%) responded to all items in some scales, and 11 (5.9%) did not respond to all items in any scale. Summaries of scores are presented in Table 3. The distributions for gender-related rejection, and nonaffirmation of gender identity were skewed to the left, indicating that participants experienced high levels of both these minority stressors. In contrast, the distribution for anticipated stigma was skewed to the right, suggesting that the sample experienced a low level of this stressor. Similarly, the distribution for community connectedness was skewed to the right, indicating that participants experienced a high level of this resilience factor. No statistically significant differences were observed between participants who reported testing for HIV or other STIs in the past year and those who did not. Also, testing behaviors did not differ between those who reported ever being forced to have sexual contact against their will and those who did not (HIV: 14.0% vs. 15.4%, Chi-square *p*-value=0.82, other STIs: 20.9% vs. 23.1%, Chi-square *p*-value=0.77).

**Table 3.** Gender-Related Stress and Resilience Levels, Overall and Stratified by Testing Behaviors in the Past Year, Reported by 186 Transgender Youth, United States, June 2017 to June 2018

		Tested for HIV in the past year			Tested for other STIs in the past year		
Scale	Overall Mean, median	Yes Mean, median	No Mean, median	*p*^a^	Yes Mean, median	No Mean, median	*p*^b^
Gender-related discrimination^c^	2.5, 2 (*N*=174)	2.3, 2 (*n*=25)	2.5, 2 (*n*=149)	0.68	2.3, 2 (*n*=38)	2.5, 2 (*n*=136)	0.57
Gender-related rejection^d^	3.6, 4 (*N*=172)	3.7, 4 (*n*=23)	3.6, 4 (*n*=149)	0.73	3.8, 5 (*n*=36)	3.6, 4 (*n*=136)	0.44
Nonaffirmation of gender identity^e^	16.8, 19 (*N*=174)	17.9, 19 (*n*=25)	16.6, 18 (*n*=149)	0.35	16.9, 18.5 (*n*=38)	16.7, 19 (*n*=136)	0.59
Internalized transphobia^f^	14.4, 14 (*N*=170)	13.0, 11 (*n*=25)	14.7, 14 (*n*=145)	0.37	15.3, 16 (*n*=37)	14.2, 14 (*n*=133)	0.44
Anticipated stigma^g^	16.4, 14 (*N*=173)	14.7, 12 (*n*=25)	16.7, 14.5 (*n*=148)	0.49	16.6, 2.5 (*n*=38)	16.4, 15 (*n*=135)	0.94
Community connectedness^h^	7.5, 7 (*N*=172)	7.1, 7 (*n*=25)	7.5, 7 (*n*=147)	0.64	6.9, 7 (*n*=38)	7.6, 8 (*n*=134)	0.63

^a^ Kruskal–Wallis tests to assess differences in gender-related stress and resilience levels across categories of testing for HIV in the past year.

^b^ Kruskal–Wallis tests to assess differences in gender-related stress and resilience levels across categories of testing for other STIs in the past year.

^c^ 5-Item scale potentially ranging from 0 to 5, with progressively increasing values indicating greater levels of experienced gender-related discrimination. Items scored 0 for never and 1 for yes: (i) had difficulty getting medical or mental health treatment, (ii) had difficulty finding a bathroom to use when out in public, (iii) had difficulty getting identity documents that match their gender identity, (iv) had difficulty finding housing or staying in housing, and (v) had difficulty finding employment or keeping employment, or had been denied a promotion.

^d^ 6-Item scale potentially ranging from 0 to 6, with progressively increasing values indicating greater levels of experienced gender-related rejection. Items scored 0 for never and 1 for yes: (i) had difficulty finding a partner, or had a relationship end, (ii) had been rejected by or made to feel unwelcome by a religious community, (iii) had been rejected by or made to feel unwelcome in their ethnic/racial community, (iv) had been rejected by or distanced from friends, (v) had been rejected at school or work, and (vi) had been rejected by or distanced from family.

^e^ 6-Item scale potentially ranging from 0 to 24, with progressively increasing values indicating greater levels of experienced non-affirmation of gender identity. Items scored 0 for strongly disagree to 4 for strongly agree: (i) have to repeatedly explain their gender identity to people or correct the pronouns people use, (ii) have difficulty being perceived as their gender, (iii) have to work hard for people to see their gender accurately, (iv) have to be “hypermasculine” or “hyperfeminine” for people to accept their gender, (v) think that people do not respect their gender identity because of their appearance or body, and (vi) think that people do not understand them because they do not see their gender as they do.

^f^ 8-Item scale potentially ranging from 0 to 32, with progressively increasing values indicating greater levels of internalized transphobia. Items scored 0 for strongly disagree to 4 for strongly agree: (i) resent their gender identity or expression, (ii) feel like a freak because of their gender identity or expression, (iii) feel depressed when they think of their gender identity or expression, (iv) feel unhappy when they think of their gender identity or expression, (v) feel like an outcast because of their gender identity or expression, (vi) often ask themselves why can't their gender identity or expression just be normal, (vii) feel that their gender identity or expression is embarrassing, and (viii) envy people who do not have a gender identity or expression like theirs.

^g^ 9-Item scale potentially ranging from 0 to 36, with progressively increasing values indicating greater levels of anticipated stigma. Items scored 0 for strongly disagree to 4 for strongly agree: If they disclosed their assigned sex at birth (i) others would not accept them, (ii) employers would not hire them, (iii) people would think they are mentally ill or “crazy,” (iv) people would think they are disgusting or sinful, (v) most people would think less of them, (vi) most people would look down on them, (vii) they could be a victim of crime or violence, (viii) they could be arrested or harassed by police, and (ix) they could be denied good medical care.

^h^ 5-Item scale potentially ranging from 0 to 20, with progressively increasing values indicating greater levels of community connectedness. Items scored 0 for strongly disagree to 4 for strongly agree: (i) feel part of a community of people who share their gender identity, (ii) feel connected to other people who share their gender identity, (iii) feel like they belong when interacting with members of the community that shares their gender identity, (iv) are not like other people who share their gender identity (reverse-scored), and (v) feel isolated and separate from other people who share their gender identity (reverse-scored).

The HIV testing characteristics of 45 participants who reported ever having been tested are described in Table 4. More than one-third (37.7%) had their most recent HIV test >1 year before the survey. Seventeen participants (37.8%) reported testing just once in the past year, and 11 (24.4%) reported testing every 6 months. Private doctors' offices were the most commonly reported location of testing (35.6%), and more than half (57.8%) indicated receiving a rapid HIV test (oral fluid or finger-stick blood). None of the participants had ever used a commercially available home HIV test. The main reasons cited included not knowing where to obtain such a test (*n*=20, 44.4%), preferring to get tested at a different location such as their doctor's office (*n*=8, 17.8%), concerns about its cost (*n*=7, 15.6%), concerns about being able to perform or interpret the test correctly (*n*=4, 8.9%), and concerns about its accuracy (*n*=3, 6.7%).

**Table 4.** HIV Testing Characteristics of 45 Transgender Youth Who Reported Ever Having Been Tested, United States, June 2017 to June 2018

Characteristic	*N*=45, *n* (%)
Time of most recent HIV test	
More than 2 years ago	6 (13.3)
Between 1 and 2 years ago	11 (24.4)
Within the past 1 year	
Tested just once	17 (37.8)
Tested every 6 months	11 (24.4)
Location of most recent HIV test	
Private doctor's office	16 (35.6)
Public health clinic/Community health center/STI clinic	12 (26.7)
Street outreach program/Mobile unit	9 (20.0)
HIV testing and counseling site	4 (8.9)
Other^a^	4 (8.9)
Type of most recent HIV test	
Test that required drawing blood with a syringe	19 (42.2)
Finger-stick blood rapid test	12 (26.7)
Oral fluid rapid test	14 (31.1)
Result of most recent HIV test	
Negative	44 (97.8)
Unknown	1 (2.2)

^a^ Includes one who reported testing in the emergency room, one who reported testing in a university clinic, one who reported testing in a private location, and one who reported testing at a conference.

Results from the multivariable logistic regression models to examine variations in testing for HIV and other STIs in the past year across categories of gender identity are summarized in Table 5. Transgender women were significantly less likely to report testing for HIV (aOR: 0.15, 95% CI: 0.03–0.78) and other STIs (aOR: 0.33, 95% CI: 0.11–0.99) compared with transgender men. In addition, participants who agreed that their health care provider is knowledgeable about transgender health issues were at least thrice as likely to report testing for HIV (aOR: 3.29, 95% CI: 1.36–7.97) and other STIs (aOR: 3.05, 95% CI: 1.40–6.63) as those who disagreed.

**Table 5.** Variations in Testing for HIV and Other Sexually Transmitted Infections in the Past Year Across Gender Identity Among 186 Transgender Youth, United States, June 2017 to June 2018

Characteristic	Tested for HIV in the past year aOR (95% CI)	Tested for other STIs in the past year^a^ aOR (95% CI)
Gender identity		
Transgender men	Reference	Reference
Transgender women	**0.15 (0.03–0.78)**	**0.33 (0.11–0.99)**
Nonbinary individuals^b^	0.63 (0.26–1.58)	0.50 (0.22–1.13)
Age group (years)^c^		
15–18	Reference	Reference
19–24	1.41 (0.40–4.99)	1.48 (0.49–4.53)
Race/ethnicity		
Non-Hispanic white	Reference	Reference
Non-Hispanic nonwhite^d^	1.20 (0.41–3.54)	0.62 (0.22–1.78)
Hispanic	1.41 (0.39–5.10)	1.53 (0.53–4.44)
Educational level		
Ninth grade to high school graduate/GED	Reference	Reference
Some college/technical school or higher	2.01 (0.58–6.95)	2.09 (0.72–6.11)
Health care provider is knowledgeable about transgender health issues^e^		
Disagree	Reference	Reference
Agree	**3.29 (1.36–7.97)**	**3.05 (1.40–6.63)**
Consumed alcohol, tobacco products, or other drugs in the past 3 months		
No	Reference	Reference
Yes^f^	0.81 (0.31–2.12)	1.05 (0.45–2.44)
No. of sex partners in the past 3 months^g^		
0	Reference	Reference
1	0.84 (0.31–2.29)	0.56 (0.23–1.32)
≥2	0.96 (0.31–2.99)	0.77 (0.30–1.98)

Values in bold indicate statistically significant results (*P* < 0.05).

^a^ Includes 34 who reported testing for gonorrhea and chlamydia, 24 who reported testing for syphilis, 22 who reported testing for genital herpes, and 22 who reported testing for human papillomavirus (numbers are not mutually exclusive).

^b^ Includes 54 who were assigned female at birth and 24 who were assigned male at birth.

^c^ Mean=19 years, median=19 years.

^d^ Includes 12 non-Hispanic black/African American, 7 Asian, 2 Native American/Alaskan Native, 1 Middle Eastern, and 17 mixed.

^e^ Responses collected as a 5-point Likert item reflecting the extent to which participants agree with this statement. Disagree includes 27 who “strongly disagree,” 48 who “somewhat disagree,” and 54 who “neither agree nor disagree.” Agree includes 22 who “strongly agree” and 35 who “somewhat agree.”

^f^ Includes 113 who consumed alcohol, 52 who used tobacco products, and 86 who used other drugs, of whom 81 reported using cannabis, 9 reported using synthetic opioids, 9 reported using amphetamines, 8 reported using cocaine, 7 reported using sedatives, 2 reported using hallucinogens, and 2 reported using amyl nitrites (numbers are not mutually exclusive).

^g^ Summarizes data from 180 who responded to questions on anal sex, 123 who responded to questions on vaginal sex, and 121 who responded to questions on oral sex.

aOR, adjusted odds ratio; CI, confidence interval.

## Discussion

Evidence is increasing that transgender individuals, particularly transgender women, in the United States are heavily impacted by HIV and other STIs.^9,12^ Adolescent and young gender minority persons might be particularly vulnerable on account of sexual experimentation and risk-taking behaviors,^25^ underscoring the importance of preventive screenings in this subgroup. Low levels of annual testing for HIV (15.1%) and other STIs (22.6%) were observed in this study of predominantly sexually active transgender youth aged 15–24 years. Furthermore, significant variations were identified across categories of gender identity, with transgender women being less likely to report testing for both HIV and other STIs compared with transgender men. Results from multivariable models suggest that low levels of testing may be driven by a lack of provider competency with transgender health care. Transgender youth are likely not being reached by existing prevention efforts, and these data highlight the exigency for developing and evaluating novel interventions for young gender minorities at risk.

Testing for HIV and other STIs is the gateway to accessing both prophylactic and therapeutic services. According to the CDC, all persons aged 13–64 years should be screened for HIV in health care settings, and those at high risk for HIV should be screened at least annually.^26^ Transgender individuals are not explicitly specified in the current HIV testing recommendations. However, the latest STI treatment guidelines recognize adolescents, transgender women, and transgender men as special populations, and recommend that screening for different STIs be performed on the basis of an assessment that considers a person's anatomy as well as patterns of potentially high-risk sexual activity.^27^ Although national estimates for the rates of screening among transgender youth are not available for comparison, it is disconcerting that less than one in six participants who recently engaged in some kind of condomless sex (anal, vaginal, or oral) reported testing for HIV in the past year, and less than one in five reported testing for other STIs. These figures parallel the low levels of testing observed among adolescent sexual minority males in the United States who report having sex without condoms,^28^ and sexually experienced youth in general.^29^ Tailored, age-appropriate strategies are urgently needed to reverse these trends.

Anatomic diversity among transgender youth is a key consideration when assessing their risk for HIV and other STIs. Although reports of gender affirmation surgeries have been increasing,^30^ many transgender persons have not yet undergone genital reconstruction procedures. In a large cohort of individuals, including transgender youth who sought gender-affirming treatments at Kaiser Permanente health plans in Georgia, Northern California, and Southern California between 2006 and 2014, 5.2% of 3,475 transgender women and 4.1% of 2,892 transgender men had undergone genital reconstruction surgery.^31^ Two participants (1.1%) in this study reported undergoing genital reconstruction surgery. Transgender women and nonbinary AMAB individuals who retain a functional penis may engage in insertive anal, vaginal, or oral sex with both men and women. Transgender men and nonbinary AFAB individuals might have a vagina and cervix, putting them at risk for bacterial (e.g., gonorrhea, chlamydia, and syphilis) and viral (e.g., HIV, genital herpes, and human papillomavirus) STIs. Only one in eight transgender women and nonbinary AMAB individuals, and one in six transgender men and nonbinary AFAB individuals in this sample reported testing for HIV in the past year. One in four transgender women and nonbinary AMAB individuals and one in five transgender men and nonbinary AFAB individuals reported testing for other STIs. Transgender youth may not feel entirely comfortable with their bodies, and might be reluctant to undergo testing, particularly in communities where gender-affirming health care services are limited.

Research indicates that transgender youth have disparately negative mental and physical health outcomes compared with their cisgender peers.^7,8^ In a recent analysis of data from the 2016 Minnesota Student Survey, 59.3% of 2,168 transgender youth endorsed having long-term mental health problems compared with 17.4% of 78,761 cisgender youth.^32^ The respective percentages for long-term physical disabilities or health problems were 25.2% and 15.2%. Poorer health outcomes in this subgroup have been hypothesized to result from experiences of social stress.^5^ Distal or external stressors include factors such as gender-related discrimination, gender-related rejection, and nonaffirmation of one's gender identity by others, whereas proximal stressors include factors such as internalized transphobia and anticipated stigma.^23,33,34^ Experiencing these stressors can result in social and economic marginalization of transgender youth^35^ and may negatively impact their access to and utilization of health care services.^36,37^ Participants in the current study experienced high levels of gender-related rejection and nonaffirmation of their gender identity, but a low level of anticipated stigma. They also experienced a high level of community connectedness, a resilience factor known to positively impact psychological well-being in sexual and gender minority youth.^38,39^ However, none of the domains was individually associated with testing for either HIV or other STIs in the past year. This is similar to results from the national Transgender Stress and Health Study conducted from 2014 to 2015 among 300 transgender adults,^40^ and suggests that other aspects might explain their suboptimal screening rates. Although physically forced sexual intercourse has been associated with higher HIV testing among adolescents,^41^ this relationship was not noted in the current analyses. One explanation is that cisgender youth might have greater access to sexual assault forensic examinations and postassault care (including prevention services) compared with transgender youth. Larger studies of transgender youth are warranted to better understand the influence of gender minority stressors and resilience factors on preventive care-seeking behaviors.

Transgender men and nonbinary individuals have been historically underrepresented in health research.^42^ Much of what is known about the risk determinants and mitigating factors for HIV and other STIs has been derived from samples of transgender women.^19^ Despite their heterogeneity, even transgender women have been conflated with men who have sex with men (MSM) in studies published as recently as 2018.^43–45^ Significant gaps in knowledge remain for transgender youth, prompting calls to engage them in future research.^46^ This study is unique in examining whether differences in testing for HIV and other STIs exist across categories of gender identity among transgender youth. Transgender women were 85% less likely to report testing for HIV in the past year compared with transgender men, after adjusting for sociodemographic factors and their number of recent sex partners. This result is particularly concerning, given the high burden of HIV among transgender women aged 13–24 years.^11^ Transgender women were also 67% less likely to report testing for other STIs compared with transgender men. These data indicate that self-reported testing levels among adolescent and young transgender women might be inconsistent with their risk profiles, further stressing the importance of customizing HIV and other STI prevention strategies to specific ages and gender identities.^47–49^

The reasons for reduced receipt of preventive screenings among transgender youth are multifactorial and may be due, in part, to the lack of access to health care providers experienced in treating gender minorities, the lack of provider awareness regarding risk assessment protocols, and screening guidelines for transgender persons, as well as the limited availability of evidence-based screening recommendations from expert organizations and professional societies.^6^ A study evaluating the lesbian, gay, bisexual, transgender, and queer (LGBTQ) content of curricula in US and Canadian medical schools found little to no education on transgender health.^50^ Participants in this study who agreed that their health care provider is knowledgeable about transgender health issues were significantly more likely to report testing for HIV and other STIs compared to those who disagreed. Although not surprising, this is an important result in light of growing consensus that health care professionals should be offered trainings in transgender-related care and cultural humility to better serve the unmet needs of this population.^51,52^ In a recent study involving 198 adolescent sexual minority males, participants who reported that their providers had initiated a discussion about their sexual orientation were significantly more likely to have received HIV and other STI preventive services and testing.^53^ Therefore, it might be possible to achieve gains in screening rates among transgender youth, especially those who engage in condomless sex, by educating health care providers about appropriate methods of initiating sexual health conversations.

Besides enhancing the delivery of health care services, attempts to improve the inclusiveness of sex education curricula in schools by emphasizing LGBTQ-specific issues could prove to be beneficial. Sexual and gender minority youth perceive the current information being provided as “exclusive” and desire a focus on HIV and other STI prevention approaches over pregnancy prevention modalities.^54^ School-based programs have been demonstrated to gradually increase the uptake of testing,^55,56^ and can engage large numbers of youth during the key developmental stages of their lives. Designing and testing new interventions to reduce sexual risks and improve screening levels is also critical to advancing HIV and other STI prevention efforts for transgender youth (TY). Rapid home HIV testing supplemented with telehealth-based counseling may be a resource efficient strategy with the potential to influence both risk-taking and care-seeking behaviors.^21^ This can also provide an opportunity to educate TY about the commercial availability of such tests, their proper conduct and interpretation, and their accuracy. Recognizing differences between people embracing various gender identities is important, and it is encouraging to note that researchers have started focusing individually on young transgender men,^57^ young transgender women,^48,58^ as well as nonbinary youth.^59^

Despite careful planning and conduct, no study is without limitations. Caution must be exercised in generalizing results to other transgender youth, as these analyses are based on a relatively small convenience sample recruited through social media. HIV testing levels noted may be lower than the general transgender youth population, because having been previously tested and diagnosed with HIV was an exclusion criterion. Black/African American transgender women, the subgroup of transgender individuals most heavily impacted by HIV in the United States,^9^ were underrepresented. Nevertheless, the low levels of annual testing for HIV and other STIs observed among sexually active participants highlight the need to engage all transgender youth in comprehensive prevention strategies, irrespective of their race or ethnicity. Some participants could have underreported their testing levels due to a long recall period, or reported a fewer number of sex partners and better than actual testing levels on account of social desirability.^60^ Finally, due to the lack of information on the timing of HIV and other STI tests relative to participants' high-risk sexual activities, it is not possible to comment regarding event-driven testing patterns.

## Conclusion

The current study helps address a significant knowledge gap regarding the prevalence of HIV and other STI testing among sexually active transgender youth. Low levels of screening, particularly among those who do not regularly use condoms, reflect a systematic failure to engage those at increased risk as part of ongoing prevention efforts. Training health care providers to better assess the sexual health status and needs of transgender youth is essential to improving the efficiency preventive screenings. Educating adolescents about HIV and other STI prevention approaches, which includes providing information that they can consent to testing without parental permission, could set the stage for lifelong protection, including those at highest risk. This is the first study to quantify testing levels within different categories of gender identity among transgender youth, and results could serve as preliminary data for proposals seeking to evaluate targeted interventions. Future prevention research should avoid conflating transgender women with MSM and prioritize transgender youth of all gender identities.

## Abbreviations Used

AFAB: assigned female at birth
AMAB: assigned male at birth
aOR: adjusted odds ratio
BRFSS: Behavioral Risk Factor Surveillance System
CDC: Centers for Disease Control and Prevention
CI: confidence interval
HIV: human immunodeficiency virus
LGBTQ: lesbian, gay, bisexual, transgender, and queer
MSM: men who have sex with men
PrEP: pre-exposure prophylaxis
STI: sexually transmitted infection
